# Congenital Hyperinsulinemic Hypoglycemia With a New HADH Mutation and Pancreatic Overexpression of GLP-1 Receptors

**DOI:** 10.1210/clinem/dgaf423

**Published:** 2025-07-25

**Authors:** Andrea Widmer, Urs Zumsteg, Gabor Szinnai, Isabel Filges, Stephanie Meier, Julie De Geyter, Kwadwo Antwi, Damian Wild, Jean-Marc Nuoffer, Emanuel Christ

**Affiliations:** Division of Endocrinology, Diabetes, and Metabolism, University Hospital Basel, Basel CH-4031, Switzerland; Division of Endocrinology, Diabetes, and Metabolism, University Children's Hospital Basel, Basel CH-4031, Switzerland; Division of Endocrinology, Diabetes, and Metabolism, University Children's Hospital Basel, Basel CH-4031, Switzerland; Medical Genetics, Institute of Medical Genetics and Pathology, University Hospital Basel, Basel CH-4031, Switzerland; Medical Genetics, Institute of Medical Genetics and Pathology, University Hospital Basel, Basel CH-4031, Switzerland; Medical Genetics, Institute of Medical Genetics and Pathology, University Hospital Basel, Basel CH-4031, Switzerland; Division of Nuclear Medicine, University Hospital Basel, Basel CH-4031, Switzerland; Division of Nuclear Medicine, University Hospital Basel, Basel CH-4031, Switzerland; Division of Laboratory Medicine, University Hospital Berne, Inselspital, Bern CH-3010, Switzerland; University Children's Hospital, University Hospital Bern, Inselspital, Bern CH-3010, Switzerland; Division of Endocrinology, Diabetes, and Metabolism, University Hospital Basel, Basel CH-4031, Switzerland

**Keywords:** congenital hyperinsulinemic hypoglycemia, HADH mutation, GLP-1 receptor imaging

## Abstract

**Background:**

The most common cause of endogenous hyperinsulinemic hypoglycemia in neonates [congenital hyperinsulinemic hypoglycemia (CHH)] is different monogenic forms of gene mutations. About 50% of the mutations are known. We present a new mutation within the short-chain L-3-hydroxyacyl-CoA dehydrogenase (*HADH*) gene causing CHH in 2 related patients.

**Methods:**

The course of 2 consanguineous patients with CHH are presented with a follow-up of >30 years. NextSeq 500 sequencing was performed and confined on known genes with autosomal recessive inheritance, namely *ABCC8* (MANE Select: NM_00352.6), *HADH* (MANE Select: NM_005327.7), and *KCNJ11* (MANE Select: NM_00525.4). Acylcarnitine profiles were measured and ^68^Ga-DOTA exendin positron emission tomography/computer tomography was performed.

**Results:**

CHH was diagnosed in both patients in the neonatal period. A therapy with diazoxid was initiated, which initially stabilized the disease in both children. With advancing age, more hypoglycemic events occurred with an increase in carbohydrate intake, leading to obesity in both patients. In addition to diazoxide, somatostatin analogues were successfully added in adulthood. A genetic analysis documented a new homozygote mutation in the *HADH* gene (HADH-variant c.796G > T). An acylcarnitine profile showed an increased plasma butyryl-carnitine, consistent with a dysfunction of the HADH enzyme. ^68^Gallium-DOTA-exendin showed an increased uptake in the whole pancreas in both patients.

**Conclusion:**

Clinical presentation, biochemical workup, and therapeutical response to diazoxide and somatostatin analogues are consistent with previous reports of HADH mutations. The overexpression of glucagon-like peptide 1 receptors in this context warrants further research.

Congenital hyperinsulinemic hypoglycemia (CHI) is the most common cause of hypoglycemia in children. The incidence of CHI varies from 1:50 000 births in Holland to 1:2500 in Saudi Arabia (1). In patients with CHI, the regulatory pathway between glucose and insulin secretion is impaired, leading to inadequate insulin secretion and consequently hypoglycemia. CHI has a high genetic and clinical heterogeneity. Monogenetic defects leading to CHI can be grouped into 4 main categories. The first group—responsible for 40% to 50% of cases—consists of defects channel transporter proteins (ABCC8, KCNJ11, KCNQ1, CACNA1D, SLC16A1). Pancreatic K-ATP channels have a critical role in the regulation of insulin release, and defects in their encoding genes cause the most prevalent and severe forms of CHI (2). The second category is enzymatic gene defects, leading to abnormalities in metabolic pathways, which converge on insulin secretion (GLUD1, GCK, HADH, UCP2, HK1, PMM2, PGM1), and the third group is related to genes encoding transcription factors (HNF1A, HNF4A, FOXA2). The fourth group includes syndromic forms. However, in about 50% to 60% of patients, the genetic cause is still unknown (3).

Currently, 3 different histological forms are known: a diffuse form with functional abnormality of islets throughout the pancreas, an atypical form where the pathophysiology is unclear, and a focal form with focal islet cell adenomatous hyperplasia, which can be cured by partial pancreatectomy. The most frequent form is the paternally inherited monoallelic K-ATP channel mutation with a postzygotic loss of the corresponding maternal region on chromosome 11, usually leading to a focal adenomatous hyperplasia accounting for 30% to 40% of the cases with CHI (3).

Glucagon-like peptide 1 (GLP-1) is a glucose-dependent insulin secretagogue and is established in the therapy of obesity with or without type 2 diabetes (4). GLP-1 receptors are in the β cells of the pancreatic islet cells and are overexpressed on the surface of benign insulin-secreting neuroendocrine tumors (insulinoma) (5). Recently, several prospective studies have shown that molecular imaging using the GLP-1 analogue exendin-4 attached to the gamma emitter ^111^Indium or the positron emitter ^68^Gallium exhibits an excellent sensitivity for the localization of benign insulinomas (6, 7). This is of importance since benign insulinoma are often small (1-2 cm), difficult to localize using conventional imaging [computed tomography (CT), magnetic resonance imaging] and do not overexpress somatostatin receptors like the other neuroendocrine tumors of the gastrointestinal tract. ^68^Ga-DOTATOC positron emission tomography (PET)/CT is, therefore, often negative in these patients. However, a reliable preoperative localization is very important to guide the surgical strategy (8).

Whether patients with nesidioblastosis overexpress GLP-1 receptors is not established. Preliminary data suggest that adult nesidioblastosis (without a history of bariatric surgery) overexpresses GLP-1 receptors and may, therefore, be a target for GLP-1 receptor imaging (8).

We report here 2 cases of first-degree cousins from a consanguineous family with CHI and the documentation of a new homozygote variant of the hydroxyacyl-CoA dehydrogenase (HADH c.796G > T). The findings of in vivo GLP-1R imaging and a follow-up of >30 years of these patients are reported.

## Material and Methods

The medical histories of these 2 patients were obtained from medical records at the University Children's Hospital Basel. The ethics committee of northwest and central Switzerland (EKNZ) confirmed a waiver for ethics permission for this project since the analysis is retrospective and not generalizable knowledge (Request-2025-00169).

### Mutation Analysis

DNA extraction was performed on EDTA blood samples (patient 1) or hair follicles/buccal swabs (patient 2). Sequence analysis of patient 1 by was by high-throughput sequencing using the library enrichment assay TruSight One expanded (Illumina) with the NextSeq 500 sequencing device (Illumina) of the coding regions, including exon/intron boundaries +/− 10 basepairs. Based on the familial history, sequence analysis was confined to known genes with autosomal recessive inheritance, namely *ABCC8* (MANE Select: NM_00352.6), *HADH* (MANE Select: NM_005327.7), and *KCNJ11* (MANE Select: NM_00525.4). Mean coverage was ca. 255x; percentage of reads with a phred score >30 was 83.2%. Alignment and variant calling used Varsome Clinical, Sentieon version: 201711.03, Thalia version: 7.0 (Saphetor). Bidirectional Sanger sequencing (primer sequences and PCR conditions available upon request) was used for confirmation of the variant.

### Measurements of Acylcarnitine Profiles

Acylcarnitine profiles were measured on a ultra-high performance liquid chromatography Aquity I-Class with Xevo TQ-S in heparin plasma and filter paper as routine analysis in the accredited metabolic laboratory of the Center of Laboratory Medicine of the University of Bern.

### GLP-1R Imaging

Synthesis and labeling of ^68^Ga-DOTA-exendin-4 have been described elsewhere (7). PET/CT was performed on a PET/128-detector CT scanner (Biograph mCT-X RT Pro Edition). One bed position of the upper abdomen was acquired during 8 minutes, 2.5 hours after IV injection of 79.8+/−3.9 MBq (range: 76-97 MBq, 12.0-15.3 mg) of 68Ga-DOTA-exendin-4. All patients underwent unenhanced low-dose CT for attenuation correction and to provide an anatomic reference (120 kVp, 30-100 mAs). All conventional scans were independently interpreted by experienced radiologists at the referral centers. GLP-1R PET/CT scans were assessed by 2 board-certified nuclear medicine physicians.

## Results

### Presentation of the 2 Cases

**Figure 1.**
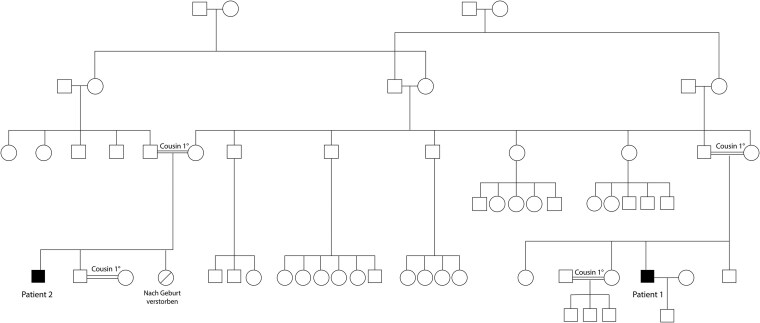
Genealogical tree.

We present a 34-year-old man (patient 1) and his 31-year-old first-degree cousin (patient 2) with CHI. They are descendants from a complex consanguineous family of Turkish origin (Fig. 1). They are cousins in the first degree by their mothers, and they are cousins in the second degree by their fathers. The respective parents of both patients are first-degree cousins.

In patient 1, the first hospitalization was at day 17 after birth because of diarrhea, emesis, and apnea with cyanosis during breastfeeding. With a blood glucose of 2.5 mmol/L and an inadequately high insulin level of 37 µE/mL, the diagnosis of hyperinsulinemic hypoglycemia of unknown etiology was made and therapy with sandostatin was initiated without achieving stabilization of blood glucose levels. Therefore, a few days later, therapy with sandostatin was stopped and therapy with diazoxide, a K-ATP-channel agonist, was initiated. Thereafter, the blood glucose levels were controlled. He had no further serious health issues in his life and had a healthy son from a nonconsanguineous relationship.

His cousin, patient 2, was hospitalized at day 10 after birth because of muscular hypotonia and sucking weakness. With a blood glucose of 2.5 mmol/L, an inadequately high insulin, and a low level of β-hydroxybutyrate, the diagnosis of hyperinsulinemic hypoglycemia was established. Therapy with diazoxide (and hydrochlorothiazide) was initiated, and normalization of blood glucose concentrations was obtained.

In contrast to his cousin (patient 1), patient 2 suffered from significant comorbidities: He was diagnosed with acute B-cell lymphocytic leukemia at the age of 26 and underwent stem cell transplantation at the age of 27 and 30. Furthermore, he was diagnosed with recurrent inflammatory syndrome of unknown cause and liver cirrhosis of unknown cause, possibly related to therapy. Liver biopsy showed florid, chronic, sclerosing steatohepatitis with severe fibrosis. Age 31, he had recurrent acute leukemia, underwent palliative chemotherapy, and died from complications of his leukemia and the associated therapy. Due to the therapeutical approach of the acute leukemia including intermittent high doses of steroids, the rare hypoglycemic events were treated symptomatically and were not in the forefront of his medical therapy. Accordingly, long-term therapy with somatostatin analogues was not established, and the hypoglycemia was mainly treated with diazoxide. Since patient 2 passed away before genetic results were available, further biochemical assessment was not possible in this case. In addition, the family refused a postmortem in patient 2; hence, histological assessment of the pancreas was not possible.

**Figure 2.**
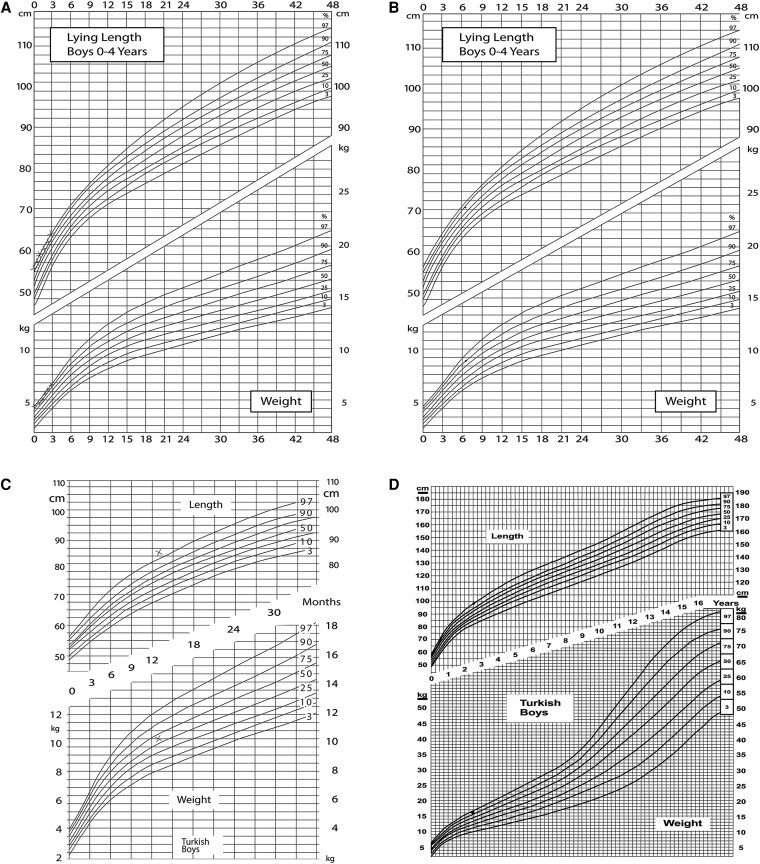
Percentile curve of patient 1 at 0 to 6 months (A), 7 months (B), 12 months (C), and 2.5 years (D).

**Figure 3.**
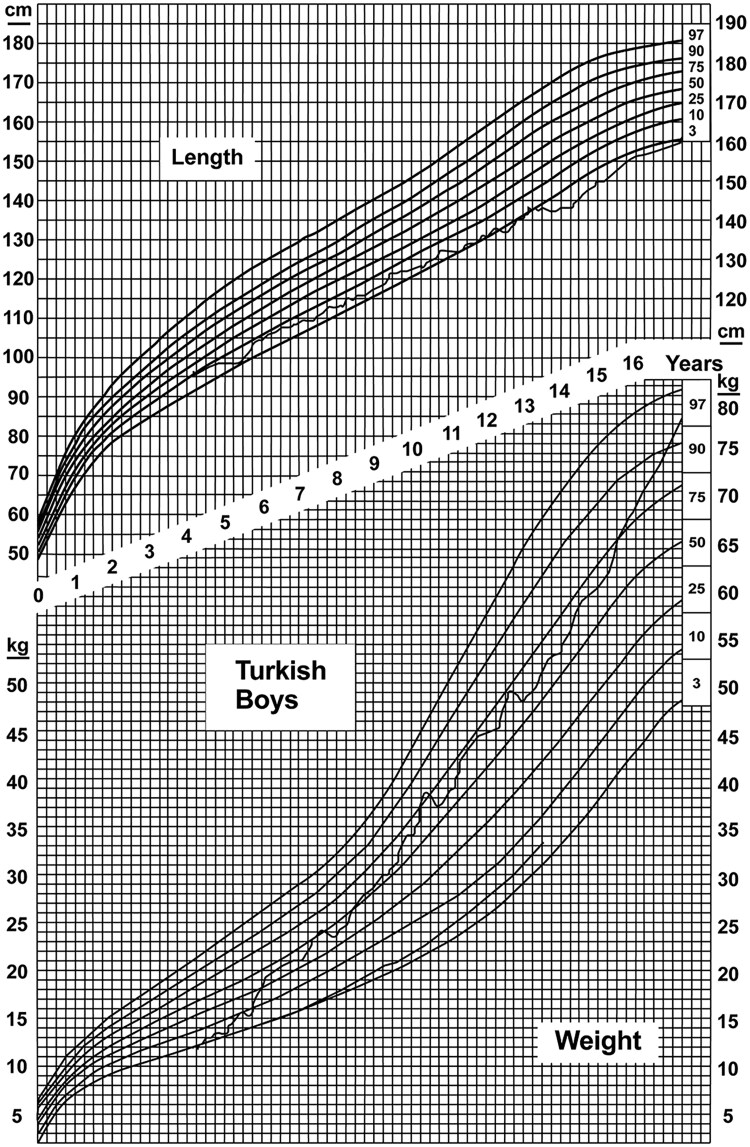
Percentile curve of patient 2 at 4 to 16 years.

The growth charts of both patients (Fig. 2 and Fig. 3) documented a disproportionate increase in weight in relation to height in childhood and adolescence, mainly due to recurrent hypoglycemic events with continuous carbohydrate snacking, both as therapy when hypoglycemia occurred and as a measure to prevent further hypoglycemias. In childhood, patient 2 was diagnosed with growth retardation, with growth documented along the 3rd percentile throughout childhood. A GH deficiency could not be excluded with certainty because of insufficient increase in the stimulation test. His final height was 165 cm. Patient 1 measured 182 cm.

In adulthood, the frequencies of hypoglycemic episodes increased, leading to the referral to the adult division of endocrinology and diabetology.

### Genetics

A novel homozygous pathogenic variant in the *HADH* gene (*HADH*-variant c.796G > T) was identified in patient 1. This variant in the penultimate exon within the C-terminal 3-hydroxyacyl-CoA dehydrogenase domain leads to a premature stop of protein synthesis in codon 266, p.(Gly266Ter), resulting in a loss of the last 49 amino acids in the short-chain 3-hydroxyacyl-coemnzyme A dehydrogenase (SCHAD) protein and has, to our knowledge, not been described in the literature yet. In accordance with the American College of Genetics and Genomics guidelines, this variant is classified as pathogenic (9-11). Criteria applied were PVS1_strong, PM2, PP3, PP4. *HADH*-related CHI follows an autosomal-recessive mode of inheritance (OMIM: *301609). Since patient 2 died in the meantime, the analysis for the presence of that variant could not be performed.

### Acylcarnitine Profile

Acylcarnitine profiles in serum and on filter paper showed repeatedly an isolated elevation of C4-OH acylcarnitine in plasma (0.27-0.43 umol/L, norm < .021) and filter paper (0.43 umol/L, norm <0.25), consistent with a dysfunction of the HADH enzyme and with the hypothesis of a new pathogenic mutation.

### GLP-1 Imaging

**Figure 4.**
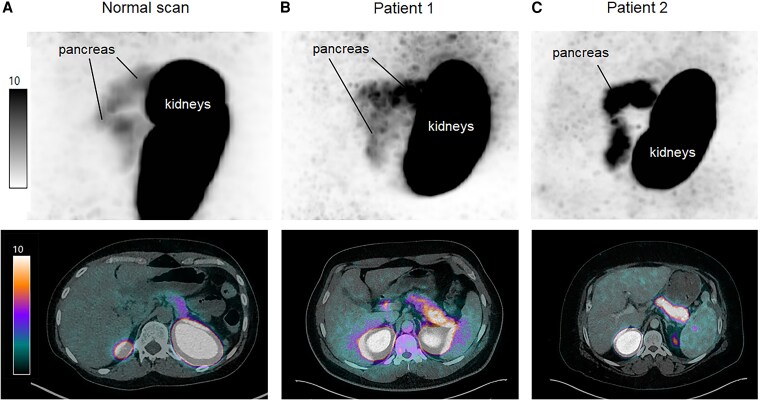
Sagittal ^68^Ga-DOTA-exendin-4 PET and transaxial ^68^Ga-DOTA-exendin-4 PET/CT from a patient with a normal scan (A), from patient 1 (B), and from patient 2 (C). Equal scale windowing was applied, with voxel values expressed in kBq. SUV_max_ of the pancreas was higher in both patients (SUV_max_ 12.3 and 20.6) compared to the patient with the normal scan (SUV_max_ 7.3). Abbreviations: CT, computed tomography; PET, positron emission tomography; SUV_max,_ maximum standardized uptake value.

^68^Gallium-DOTA-exendin PET/CT showed increased uptake in the whole pancreas (maximum standardized uptake value 12.3 and 20.6) in both patients compatible with a diffuse form of nesidioblastosis (Fig. 4A and 4B). Physiological pancreas uptake was lower with a median (range) maximum standardized uptake value of 8.0 (6.5-10.3) in 20 consecutive patients with endogenous hyperinsulinemic hypoglycaemia.

### Therapy

**Figure 5.**
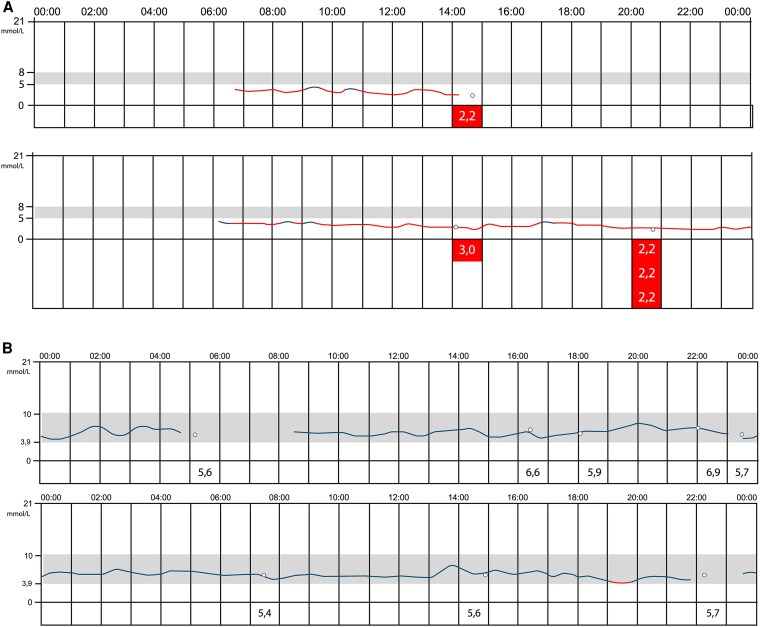
Continuous glucose monitoring system of patient 1 without sandostatin (proglicem only) (A) and with sandostatin (and proglicem) (B).

**Figure 6.**
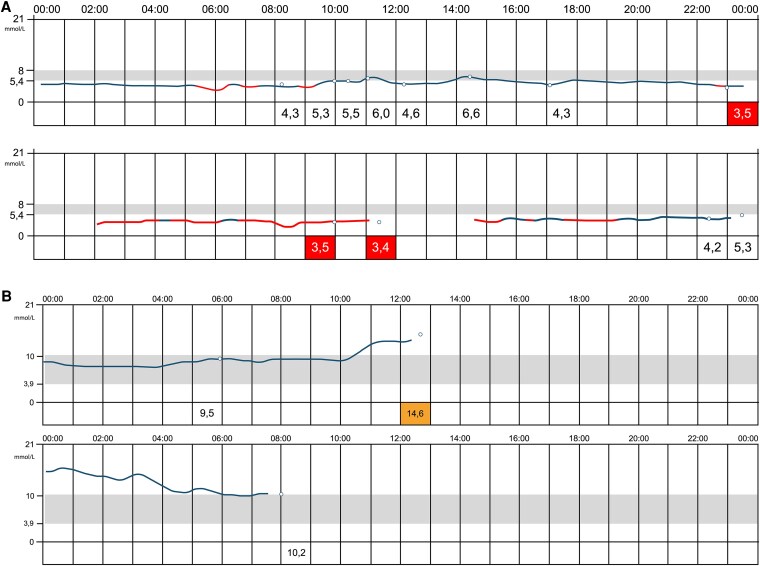
Continuous glucose monitoring system of patient 2 without sandostatin (proglicem only) (A) and with sandostatin (and proglicem) (B).

We provided both patients with a continuous glucose monitoring system, which showed daily hypoglycemia (partially also severe hypoglycemia; Figs. 5A and 6A). In addition to diazoxide long acting release octreotide was administered, which resulted in a significant decrease in hypoglycemic events (Fig. 5B and Fig. 6B). Due to significantly less hypoglycemia and therefore a decrease in snacking, patient 1 lost 24 kg within 9 months.

## Discussion

We describe a novel homozygous pathogenic variant in the *HADH* gene (*HADH*-variant c.796G > T) coding for the SCHAD protein, biochemically confirmed by a pathological acylcarnitine profile. This mutation led to neonatal CHI. Interestingly, in both patients, a diffuse overexpression of GLP-1 receptors in the pancreas could be documented by ^68^Ga-DOTA exendin PET/CT.

The fact that these 2 patients had normal birth weight and clinical and biochemical confirmed CHI in early infancy is consistent with clinical data in the literature of patients with SCHAD-CHI (12). Furthermore, the impaired acylcarnitine profile in patient 1, characterized by an increased plasma 4-hydroxybutyryl-carnitine concentration, suggesting impaired fatty oxidation, is consistent with a dysfunction of the SCHAD protein, initially described by Clayton et al (13). Mutation in the *HADH* gene is a rare condition and accounts for only about 1% of mutations leading to CHI (14). So far, approximately 25 mutations in approximately 45 patients have been described (12). The SCHAD protein is a mitochondrial enzyme that catalyzes the penultimate step in fatty acid oxidation (12). Importantly, although SCHAD protein is expressed in multiple tissues, the highest level of expression is documented in pancreatic β cells (12), indicating that it may play an important role in insulin secretion. Although the exact underlying molecular mechanism is ill-defined, a direct protein-protein interaction between SCHAD protein and glutamate dehydrogenase (GDH) is postulated (15), leading to increased activity of glutamate dehydrogenase. This, in turn, increases intracellular ATP production, thereby closing the ATP-sensitive potassium channels and consequently resulting in a calcium influx into the β cell, which activates autonomous insulin secretion (15). The role of HADH mutations in the pathophysiology of CHI is further substantiated by the HADH knockout mice model. These mice present with decreased glucose and increased insulin levels when compared with wild-type mice (16). Usually patients with SCHAD-CHI do not undergo pancreatectomy. Therefore, histological assessment in these patients is scarce. However, all pancreatic samples reported from patients with SCHAD-CHI show diffuse disease of the whole pancreas (17). This is consistent with the current finding of a potentially diffuse disease of the whole pancreas. Treatment strategies for SCHAD-CHI include—in the absence of structural changes of the K-ATP channel—diazoxide, a K-ATP channel activator, and second-line somatostatin analogues. In keeping with the literature (18, 19), our patients were responsive to these therapies as shown in Fig 5A and 5B.

The finding of a diffuse overexpression of GLP-1 receptors in the pancreas of our patients is intriguing. In clinical practice, ^18^F-DOPA-PET is the gold standard in CHI to distinguish between focal or diffuse disease (20), and this modality is superior to molecular imaging targeting somatostatin receptors such as ^68^Ga-DOTATATE (20). We (8) and others (21) have shown that ^68^Ga-DOTA exendin-4 PET/CT is a very sensitive tool to localize benign insulinomas. The molecular background is that benign insulinomas have an approximately 5-fold higher GLP-1 receptors expression compared with the normal β cell (5). We can only speculate about the mechanisms linking CHI to GLP-1 receptors overexpression. The following evidence may explain the finding: HADH-mutation results in an increased insulin secretion as mentioned before (15). A chronic increase in autonomous insulin secretion may result in an increase in the cAMP/PKA-signaling cascade, which activates GLP-1 receptor expression via c-AMP-response element binding protein (22). In addition, an increased chronic insulin secretion may lead to an inhibition of forkhead box O1, which is known to be a negative regulator of GLP-1 receptor expression (23).

## Conclusion

We report here a novel homozygous pathogenic variant in the *HADH* gene (*HADH*-variant c.796G > T) in 2 consanguineous patients. Clinical presentation and biochemical workup are reported. In addition, a long-term follow-up (>30 year) is described, and the therapeutical response to diazoxide and somatostatin analogues is documented. The overexpression of GLP-1 receptors in this context warrants further research but may be related to the insulin-induced GLP-1 receptor upregulation mediated by c-AMP-response element binding protein and/or forkhead box O1.

## Data Availability

Original data generated and analyzed during this study are included in this published article or in the data repositories listed in References.
